# Evidence for asymmetrical hybridization despite pre- and post-pollination reproductive barriers between two *Silene* species

**DOI:** 10.1093/aobpla/plw032

**Published:** 2016-07-11

**Authors:** Jin-Ju Zhang, Benjamin R. Montgomery, Shuang-Quan Huang

**Affiliations:** ^1^School of Life Sciences, Central China Normal University, Wuhan 430079, China; ^2^State Key Laboratory of Hybrid Rice, College of Life Sciences, Wuhan University, Wuhan 430072, China; ^3^Division of Natural Sciences & Engineering, University of South Carolina Upstate, Spartanburg, SC 29303, USA

**Keywords:** Asymmetric hybridization, fecundity, floral phenology, floral traits, pollinator preference, *Silene*, species integrity

## Abstract

Interspecific hybridization is widespread among plants; nevertheless, pre- and post-zygotic isolating mechanisms may maintain species integrity for interfertile species in sympatry despite some gene flow. Interspecific hybridization and potential isolating barriers were evaluated between co-flowering *Silene asclepiadea* and *Silene yunnanensis* in an alpine community in southwest China. We investigated morphological and molecular (nuclear microsatellites and chloroplast gene sequence) variation in sympatric populations of *S. asclepiadea* and *S. yunnanensis.* Additionally, we analyzed pollinator behaviour and compared reproductive success between the putative hybrids and their parental species. Both the molecular and morphological data indicate that there were putative natural hybrids in the field, with *S. asclepiadae* the ovule parent and *S. yunnanensis* the pollen parent. Bumblebees were the primary visitors to *S. asclepiadae* and putative hybrids, while butterflies were the primary visitors to *S. yunnanensis*. Pollen production and viability were significantly lower in putative hybrids than the parental species. The direction of hybridization is quite asymmetric from *S. yunnanensis* to *S. asclepiadea*. Protandry combined with later peak flowering of *S. yunnanensis*, and pollinator preference may have contributed to the asymmetric pattern of hybridization, but putative hybrids were rare. Our results thus suggest that despite gene flow, *S. asclepiadea* and *S. yunnanensis* can maintain species boundaries, perhaps as a result of floral isolation and low fecundity of the hybrids.

## Abstract (precis)

Co-flowering species may undergo interspecific hybridization if they are closely related and share pollinators. However, a series of reproductive barriers between species can prevent interspecific gene flow, making natural hybridization a transient, rare event. Both morphological and molecular data indicated putative natural hybrids between two Silene species from southwest China, with pollen from *S. yunnanensis* fertilizing ovules of *S. asclepiadae*. Zhang et al. found that pollen production and viability were significantly lower in putative hybrids than the parental species. The low fecundity of the hybrids and other reproductive barriers between the two species could contribute to species fidelity.

## Introduction

Hybridization may allow for interspecific gene flow, which at high rates can erode species integrity. Hybridization may be common, and it has been suggested that 25 % of plant species hybridize with other species (Mallet 2005), though the number of well-documented cases of natural hybridization is much less than expected (Brennan *et al.* 2012). Despite the potential for hybridiation, a series of reproductive barriers may prevent interspecific gene flow (Baack *et al.* 2015), making natural hybridization a transient, rare event. For example, flowering plants in sympatry may employ different pollinators, or the same pollinator in different flowering periods, or differ in placement of pollen on the same pollinator’s body to reduce interspecific pollen flow (Huang and Shi 2013). The role of pollinator-mediated floral isolation has been questioned given that pollinator inconstant visits may result in interspecific pollen flow, resulting in hybridization and introgression (Armbruster 2014). Therefore, studies on the coexistence of parents and hybrids can help us to understand mechanisms that maintain species boundaries.

The ability of first-generation hybrids to backcross with parental species depends on breeding systems, patterns of pollen transfer among hybrids and the parental species, as well as the viability and fertility of further generation offspring (Burke and Arnold 2001; Grant 1993). Asymmetrical sharing of pollinators between the hybrid and one parental species could contribute to asymmetrical patterns of introgression (Field *et al.* 2011; Tiffin *et al.* 2001). However, pollinators are usually generalists which may not distinguish between hybrids and parents, promoting gene exchange between parental species and hybrids (Chung *et al.* 2005; Haselhorst and Buerkle 2013).

The relative fitness of hybrid progeny is another important factor in determining the fate of hybrids (Burke and Arnold 2001). Hybrids may exhibit a reduction in growth or survival as a result of genetic incompatibilities or the breakup of coadapted gene complexes (Demuth and Wade 2005; Leinonen *et al.* 2011). Lower fitness of hybrids accompanied by persistent gene flow may result in a stable hybrid zone (Barton and Hewitt 1985). Alternatively, increased fitness in hybrids may allow them to compete successfully with the parents, in extreme cases resulting in the extinction of one of the hybridizing taxa via genetic assimilation (Hegde *et al.* 2006). Backcrossing between hybrids and parental species allows for introgression (Magnussen and Hauser 2007; Ma *et al.* 2014). Therefore, in order to assess the role of hybridization in the evolution of interfertile species, it is important to determine the fitness of hybrids as well as behaviour of pollinators towards hybrids and parental species.

*Silene* (Caryophyllaceae) is a diverse plant genus comprising over 700 species, *sensu lato* (Greuter 1995; Jenkins and Keller 2011). The genus is widely distributed in Europe, Asia and North America and has colonized a wide range of habitats, ranging from riverbanks to heavy metal polluted soils, and even frozen plateau (Greuter 1995; Jenkins and Keller 2011). Most species are annual to perennial herbaceous plants, and the genus exhibits substantial diversity especially with regard to life cycles, pollination syndromes, and breeding systems (Bernasconi *et al.* 2009; Greuter 1995; Jenkins and Keller 2011). *Silene* has become a model system for understanding the evolution of sexual systems and sex chromosomes (Blavet *et al.* 2012; Demuth *et al.* 2014; Lengerova *et al.* 2003), speciation and reproductive isolation (Karrenberg and Favre 2008; Muir *et al.* 2012), and the effects of nursery pollinators on pollination and seed predation (Kephart *et al.* 2006). Additionally, studies have investigated interspecific hybridization and isolating mechanisms, primarily focusing on *Silene* *latifolia* and its relatives (Brothers and Atwell 2014; Goulson and Jerrim 1997; Karrenberg and Favre 2008; Minder *et al.* 2007; Montgomery *et al.* 2010; Prentice 1978; Rahmé *et al.* 2009).

In China, *Silene* includes about 110 species, with 67 species endemic, mainly distributed in the northwest and southwest of China (Zhou *et al.* 2001). Our field survey in southwestern China showed several *Silene* species generally co-occurred within 1 km^2^ area. The two native species, *Silene yunnanensis* and *Silene* *asclepiadea* naturally co-occur in Shangri-La county, northwestern Yunnan Province, China. Our initial investigation observed putative hybrids with fruit and flower morphology intermediate between the two species (B. Montgomery and S.-Q. Huang, unpubl. data).

To assess the degree of hybridization and introgression, accurate identification of hybrid genealogies is necessary. Classification of species has been mainly based on morphological characters; however, diagnostic (species-specific) features discriminating between taxa are often limited (Rieseberg and Ellstrand 1993; Vereecken *et al.* 2010). The use of molecular genetic markers has avoided some of the limitations of morphological characters. Microsatellite (simple sequence repeat, SSR) makers were chosen due to their codominance and ease of identification in studies of hybridization. Molecular markers from organelle genomes (e.g. chloroplasts), which typically have maternal inheritance and low recombination rates, can be used to elucidate the direction of interspecific gene flow and gene introgression that might not be detected by nuclear markers (Bleeker and Hurka 2001). In this study, we evaluated hybridization and introgression in sympatric populations of *S. asclepiadea* and *S. yunnanensis* based on morphological and molecular (SSR and chloroplast gene sequencing) data, and we investigated the roles of pollinator preference and diminished hybrid fecundity in creating pre- and post-zygotic barriers to hybridization, respectively.

## Methods

### The study species

**Figure 1.**
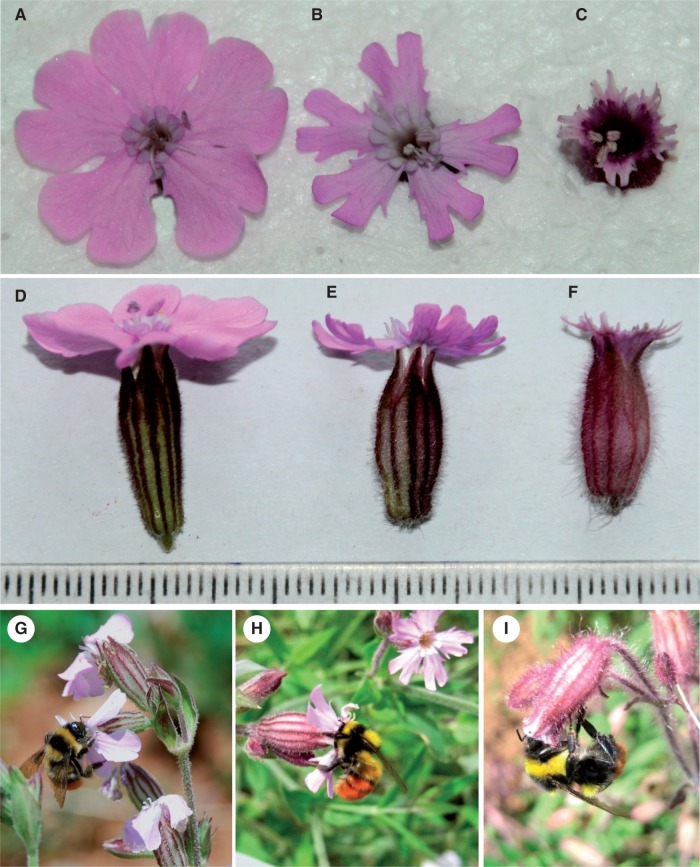
Flowers and a shared bumblebee pollinator of the two *Silene* species and a putative hybrid. An apical and side view of *S. yunnanensis* **(A, D)**, hybrid **(B, E)** and *S. asclepiadea* **(C, F)** flower; and *Bombus festivus* sucking nectar from flowers of the three taxa **(G–I),** respectively. Minimum scale at second row = 1 mm.

The study was conducted in a university field station, Shangri-La Alpine Botanical Garden (SABG, 27°54′ N, 99°38′ E) of Yunnan Province, southwest China (see Fang and Huang 2013). The two studied species, *S.* *asclepiadea* Franchet and *S.* *yunnanensis* Franchet, are closely related within the ‘Cucubaloideae’ group (Zhou *et al.* 2001). Both species are perennial herbs that co-occur naturally in open alpine meadows mixed with shrubs and near the forest edges in the study site. Geographic ranges overlap, with *S.* *yunnanensis* reported from northwest Yunnan Province and *S.* *aspclediadea* from Yunnan and two neighboring provinces (eFloras 2008). The two species are protandrous, primarily distinguished by the flower and fruit morphology. Flowers of *S. yunnanensis* are pink with narrow, long, calyx tubes; its petal-limb length (the portion of the petal above the fused calyx) is much greater than that of *S. asclepiadea*, which has flowers with dark pink petals and short, broad calyx tubes (Fig. 1). *S.* *asclepiadea* flowers earlier (early July) than *S. yunnanensis* (middle July), but their flowering periods overlap several weeks in SABG, permitting potential interspecific hybridization. Fruit diameters of *S. asclepiadea* are broader than *S. yunnanensis*. Dozens of flowering individuals each of the two species were observed in one 50 m × 50 m meadow, suggesting that the population densities of both species were high.

### Morphometric measurement and crossing experiments

To determine if putative hybrids are morphometrically intermediate between the parental species, floral and fruit characters were measured from 30 individuals from both parent species and 11 individuals of putative hybrids, respectively. Five diagnostic morphological traits were measured with a digital caliper, including calyx tube length, calyx tube diameter, corolla diameter, fruit length and fruit diameter. Flowers at the male-phase were measured on live plants in July 2012. All morphological data were analyzed using principal coordinate analysis (PCA) in STATISTICA (StatSoft 2012).

Reciprocal crosses were performed to evaluate the interfertility of *S. asclepiadea* and *S. yunnanensis*. We performed treatments with 10 individuals per species using three or four randomly selected flowers per plant. Flowers of both species were emasculated prior to anthesis and bagged with 1 mm mesh nylon tulle to exclude pollinators. Fresh pollen from each species was transferred manually to the stigma of other species and bagged again until the flower completely withered.

### Molecular markers

Leaf material was collected from flowering individuals. A total of 37 *S. asclepiadea*, 40 *S. yunnanensis*, and 11 putative hybrids were collected. Samples were placed in individually marked bags and stored in silica gel desiccant.

DNA extraction and SSR analysis: Genomic DNA was extracted from silica-dried leaf material with the Cetyl Trimethyl Ammonium Bromide method (Doyle and Doyle 1987). Twenty-four microsatellite markers developed by Juillet *et al.* (2003) and Moccia *et al.* (2009) were used for genotyping ten individuals to assess polymorphism. Of the 24 primer pairs tested, 14 successfully amplified the target regions, and 8 (SL-eSSR02, SL-eSSR08, SL-eSSR12, SL-eSSR13, SL-eSSR16, SL-eSSR17, SL-eSSR26 and A11) revealed microsatellite polymorphism. Thus, these eight polymorphic microsatellites were used to genotype all of the studied individuals. Polymerase chain reaction (PCR) was performed in a 10 μl volume, containing 25 ng genomic DNA, 0.25 μM of each primer, 0.2 mM of each dNTP, 1× buffer, 1.5 mM MgCl_2_ and 0.5 U Taq DNA polymerase. Amplifications were conducted in an ABI (Applied Biosystems) Thermocycler. Using the following parameters: 5 min at 95 °C, 35 cycles composed of 50 s denaturing at 94 °C, 50 s annealing at 56 °C, 60 s extension at 72 °C, and a final extension at 72 °C for 10 min. PCR products were amplified with the 5’ end of one primer of each primer pair labelled with a fluorescent dye, either 6-FAM (SL-eSSR02, SL-eSSR08, SL-eSSR 13, SL-eSSR16) or HEX (SL-eSSR12, SL-eSSR17, SL-eSSR26, A11). Two PCR products of different loci were multiplexed according to the fluorescent dyes and s differences, and separated by capillary gel electrophoresis on an ABI 3730xl automated sequencer (Applied Biosystems). Microsatellite allele sizes were determined with GeneMapper software 4.0 (Applied Biosystems).

### Types of hybrids between *S. asclepiadea* and *S.* *yunnanensis*

A Bayesian algorithm implemented in the software STRUCTURE version 2.3.4 (Hubisz *et al.* 2009) was used to analyze the species status of *S. yunnanensis* and *S. asclepiadea* and to detect interspecific hybrids. We tried different values of *K* from 1 to 10, and for each *K*, 5 runs were performed. The appropriate number of clusters was estimated using Evanno’s Δ*K* parameter (Evanno *et al.* 2005) using the STRUSTURE HARVESTER programme (Earl and von Holdt 2012). Markov’s chain Monte Carlo (MCMC) simulation parameters were set for a burn-in period of 50,000 and a run length of 10^6^ iterations under the model of population admixture and the assumption that allele frequencies were correlated within populations. We conducted 10 independent runs for testing robustness of the applied model to ensure consistent results.

PCA was used to determine whether hybrids existed between the two species. PCoA was performed on the distance matrices of squared Euclidean distances between all pairs of genotypes by using GENALEX 6.1 (Peakall and Smouse 2006).

### Chloroplast haplotypes and gene introgression

We sampled a total of 31 individuals, including 11 putative hybrids and 10 individuals of each parental species. Four non-coding regions of the cpDNA were amplified for two species using the following primer pairs: *trnL*-e/*trnL*-f (Taberlet *et al.* 1991), *psbA*/*trnH* (Sang *et al.* 1997), *atpB*/*rbcL* (Chiang *et al.* 1998) and *trnS*/*trnG* (Hamilton 1999). Of the four primer pairs screened, *psbA/trnH* and *atpB*/*rbcL* were found to be the most variable and were therefore selected for genotyping all 31 individuals. Amplification and sequencing followed protocols in Chiang *et al.* (1998) and Sang *et al.* (1997), respectively.

The cpDNA sequences were initially aligned using the CLUSTAL_X (Thompson *et al.* 1997) and then refined manually. All sequences of *psbA/trnH* and *atpB*/*rbcL* have been deposited in GenBank under accession numbers KT724286-KT724293.

### Phenology

We monitored the phenology of *S. asclepiadae* and *S. yunnanensis* from 12 July to 10 August, 2012, and we monitored the phenology of hybrids from 29 July to 10 August. For both species and hybrids, we haphazardly selected 12 plants from across several patches and up to 4 stems per plant, as available. We counted the number of previously opened flowers that had already senesced upon initial surveys, then surveyed selected stems of each plant every 3 or 4 days thereafter, counting the number of open flowers. On the final survey date, we counted remaining floral buds. To create cumulative flowering curves, we calculated the total number of open flowers surveyed across all observations, including senesced flowers and remaining buds, and determined the percentage of this total count that had opened by each survey date.

### Pollinator observations and insect visitation rate

To measure pollinator visitation rates, we haphazardly selected a patch of flowering plants and counted the numbers of open flowers. We recorded every floral visitor to parental species and putative hybrids each for 20 observation periods. On average, there were 75.0 ± 4.2 (range 42–105) flowers of *S. asclepiadea*), 56.5 ± 4.6 (26–120) flowers of *S. yunnanensis* and 48.3 ± 2.8 (28–75) flowers of putative hybrids in one observed patch. We observed floral visitors randomly in 12 clear days from 10.00 a.m. and 18.00 p.m. of local time when pollinators were active with each 30-min per period. Pollinators were defined as visiting a flower when they contacted the stigma or anthers. In addition, we observed 10 h during 20:00–23:00 in 4 clear days to examine moth pollinators, but none were observed visiting these species.

### Measurements of pollen and ovules

For each species, 30 flowers were collected just prior to anther dehiscence. The anthers were lacerated and squeezed to release pollen in a 1 ml aniline blue/lactophenol solution. The solution was vortexed, and pollen grains were counted for 10 aliqots, each 10 µl, under a compound microscope. The numbers of uniformly dark stained (viable) and unstained (non-viable) grains were counted, respectively. Ovules were fixed in 70 % ethanol. All ovules were counted under the dissecting microscope. We estimated the ratio of pollen/ovule (P/O) and viable P/O for each flower.

### Measurements of fruit and seed production

Fruit set and seed set for naturally pollinated flowers were measured for 30 flowering individuals of both parental species and all putative hybrids. Once flowering ceased and fruits ripened, inflorescences and fruits were counted and one to three fruits per plant were sampled to estimate seed production. Fruit set was defined as the per plant proportion of capsules produced per flower. Seed set was calculated as fully formed seeds divided by the total number of fully developed seeds, and incompletely developed or undeveloped ovules. The average number of seeds per fruit was then calculated for each plant based on seed number in sampled fruits. For each selected capsule, we weighed the seed mass. Fruits exhibiting evidence of seed predation were removed from analysis of percentage seed set and number of seeds.

All measures were compared between parental species and hybrids using one-way analysis of variance using SPSS version 19.0.

## Results

### Species identities and crossing experiments

**Figure 2.**
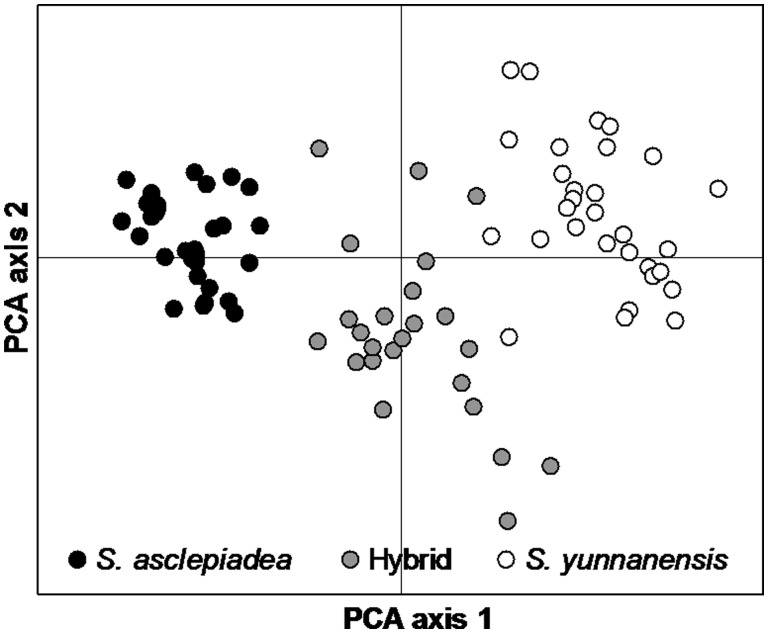
Two-dimensional plot of the PCA showing morphological relatedness between *S. asclepiadea*, putative hybrids and *S. yunnanensis* based on 5 morphological measures (calyx tube length, calyx tube diameter, corolla diameter, fruit length and fruit diameter). Axes 1 and 2 explain 34.81 and 20.96 % of the total variance, respectively.

**Figure 3.**
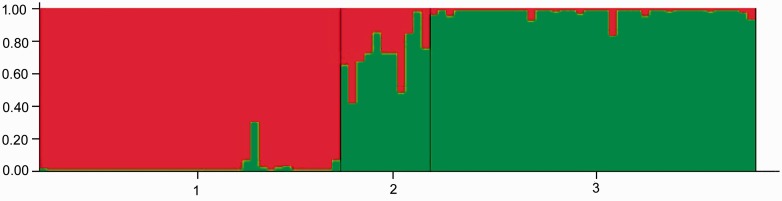
Bayesian probability of assignment of 88 individuals to a cluster (*K* = 2) on the basis of multilocus microsatellite genotypes. Populations 1, 2 and 3 are from *S. asclepiadea*, putative hybrids and *S. yunnanensis*, respectively.

For PCA of morphological measures, principal coordinate axes 1 and 2 explained 34.81 % and 20.96 % of the variation, respectively, and revealed that adult individuals belonged to three separate clusters, corresponding to each parental species and a cluster of hybrids (Fig. 2). The morphological groupings were congruent with the individuals identified as being hybrids by the analysis of microsatellite polymorphisms. Evanno’s Δ*K* was maximum when *K* = 2, suggesting assignment of all individuals to two clusters. The analysis indicated a high degree of purity of both parental species, with a membership proportion of the *S. asclepiadea* population (population 1) in the *S. asclepiadea* cluster of 97.7 %, and a membership proportion of the *S. yunnanensis* population (population 3) of 97.9 % in the *S. yunnanensis* cluster (Fig. 3). The analysis did not resolve the hybrids into a distinct cluster; instead, all hybrid individuals were assigned to either *S. asclepiadea* population (28.5 %) or the *S. yunnanensis* population (71.5 %), However, 10 of 11 individuals putatively identified as being hybrids based on morphology had microsatellite genotypes strongly suggestive of contributions from both parent species; this was not the case for most individuals identified as belonging to the parent species based on morphology (Fig. 3).

**Figure 4.**
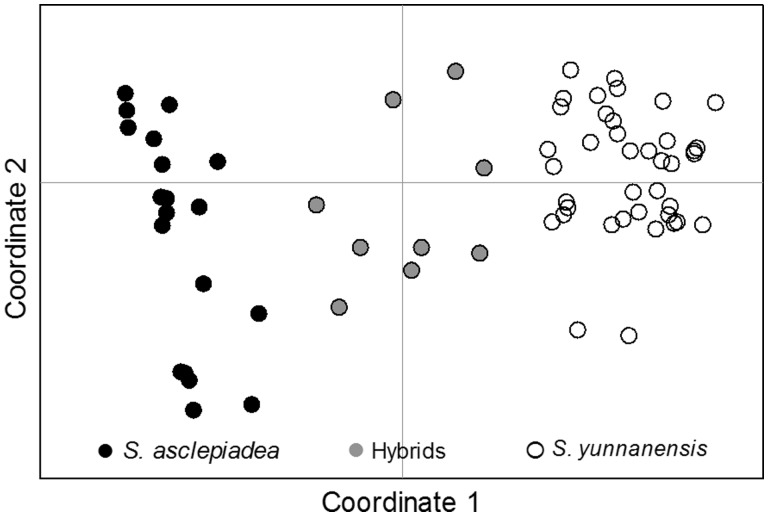
PCA showing genetic relatedness among the studied individuals. Axes 1 and 2 explain 36.80 and 20.68 % of the total variance, respectively.

For PCoA of the SSR data, principal coordinate axes 1 and 2 explained 36.80 and 20.68 % of the variation, respectively, and revealed that all studied individuals belonged to three separate gene pools (Fig. 4).

Interspecific pollinations were successful and hybrid seeds were obtained from reciprocal crosses between *S. yunnanensis* and *S. asclepiadea*. Fruit set was 87.50 % (28/32, i.e. 28 fruits yielded from 32 cross-pollinated flowers) for *S. asclepiadea* flowers and 87.10 % (27/31) for *S. yunnanensis* flowers. Five fruits were broken by haymaking for *S. asclepiadea* and 17 fruits were herbivorized for *S. yunnanensis*. In *S. asclepiadea*, about 62.66 % of ovules developed into mature seeds and a fruit produced an average of 53.74 ± 5.12 (mean ± SE) mature seeds (*n* = 23 fruits). In *S. yunnanensis*, about 52.42 % of ovules developed into mature seeds and a fruit produced 43.70 ± 5.20 mature seeds (*n* = 10). Given that higher rates of herbivory in *S. yunnanensis* mother treatments prevented us from directly contrasting total seed production per flower between the two treatments, we counted the matured seeds (initial) from young fruits before larvae damage.

### Genetic diversity, chloroplast haplotypes and gene introgression

The eight microsatellite markers we used were highly polymorphic (2–10 alleles per locus and an average value of 5.1, Table 1). Of 37 *S. asclepiadea* and 40 *S. yunnanensis* plants examined, 24 unique alleles were detected in *S. yunnanensis* but only 6 unique alleles were detected in *S. asclepiadea*. Of the 24 alleles unique to *S. yunnanensis*, eight were also present in the putative hybrids. With respect to *S. asclepiadea*, of six unique alleles, four were present in putative hybrids (Table 1). We did not observe any allele in the putative hybrids that was absent in both parental taxa.

**Table 1.** Characterization and utility of eight microsatellite markers for adult individuals of *S. yunnanensis* (n = 37), *S. asclepiadea* (*n* = 40) and putative hybrids (*n* = 11), respectively collected from Shangri-La Botanical Garden of Yunnan Province. A, number of alleles; n, sample size.

Locus	A	*n*	Allele size (bp) *S. asclepiadea*	Allele size (bp) *S. yunnanensis*	Allele size (bp) all putative hybrids
SL-eSSR02	4	88	200	200, 205, 212, 215	200
SL-eSSR08	8	88	210, 216, 222, 231	210, 219, 222, 225, 228, 231, 237	210, 216, 219, 225, 228, 231
SL-eSSR12	6	88	149, 151, 152	149, 151, 153, 155, 157	152, 155, 157
SL-eSSR13	5	88	182, 190, 196, 200	178, 182, 190, 200	182, 190
SL-eSSR16	2	88	164, 165	164, 165	164, 165
SL-eSSR17	10	88	192, 193, 197	196, 197, 199, 200, 202, 204, 206, 207	192, 193, 196, 204, 206,
SL-eSSR26	2	88	160, 198	160, 198	160, 198
A11	4	88	132, 140, 146, 150	132, 146, 150	132, 146, 150
Total	41	88			
Average	5.1				

A total of five chloroplast DNA haplotypes were detected (Table 2). *S.* *asclepiadea* possessed haplotype H1, H2 and H3, and *S. yunnanensis* had haplotype H4 and H5. The two species did not share haplotypes with each other. Among the putative hybrids, only one individual had haplotype H1, and all others had haplotype H3.

**Table 2.** Variable nucleotide sites and length polymorphisms cpDNA (*psbA/trnH* and *atpB*/*rbcL*) sequences in *S. asclepiadea*, *S. yunnanensis* and putative hybrids, respectively. Dashes (–) denote indel.

Haplotype	Species (numbers)	*psbA/trnH* variable sites											*atpB*/*rbcL variable sites*		
		70	77-85	140	175-178	206	211	219-240	266	271	285	340	87	146-149	591-598
H1	*S. asclepiadea* (1)	–	– – – – – – – – –	–	ATTT	A	C	– – – – – – – – – – – – – – – – – – – –	G	G	–	G	A	GGTT	CACATAA
	Hybrid (1)														
H2	*S. asclepiadea* (2)	–	TTTGAAAGT	–	ATTT	A	C	– – – – – – – – – – – – – – – – – – – –	G	G	–	G	A	GGTT	CACATAA
H3	*S. asclepiadea* (7)	–	TTTGAAAGT	–	ATTT	A	C	– – – – – – – – – – – – – – – – – – – –	G	G	–	G	A	GGTT	– – – – – – –
	Hybrid (10)														
H4	*S. yunnanensis* (3)	–	TTTGAAAGT	C	– – – –	–	G	ACAAAAGTCTTATTATGTCAAT	A	G	T	–	–	– – – –	– – – – – – –
H5	*S. yunnanensis* (7)	T	TTTGAAAGT	C	– – – T	A	C	ACAAAAGTCTTATTATGTCAAT	A	A	T	G	–	GGTT	– – – – – – –

### Phenology

**Figure 5.**
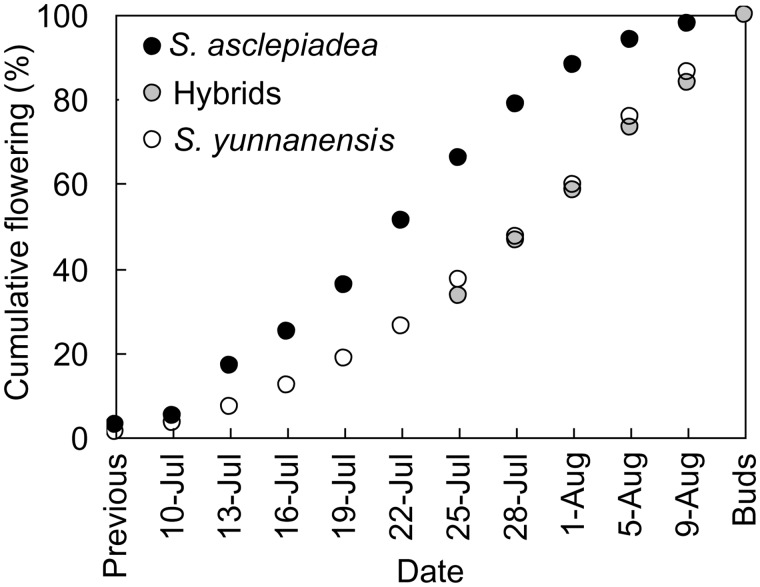
Cumulative flowering curves for *S. asclepiadea* and *S. yunnanensis* at Shangri-La Botanic Garden, Yunnan Province, China, based on up to 4 flowering stems from each of 12 plants per species, and including flowers already senesced previous to first survey date (previous) and buds remaining on final survey date (Buds). For hybrids, the first survey date was 29 July, and flowers senesced previously are represented as the value for 26 July.

Flowering curves overlapped for both parental species and putative hybrids (Fig. 5). However, cumulative flowering curves for *S. asclepiadea* were higher than *S. yunnanensis* for all survey dates, indicating that on average flowering occurred earlier for *S. ascplepiadea*. The date by which 50 % of flowering was observed for *S. asclepiadea* was 23 July compared with 2 August for *S. yunnanensis* and hybrids, a difference of 10 days.

### Pollinator observations and insect visitation rate

**Figure 6.**
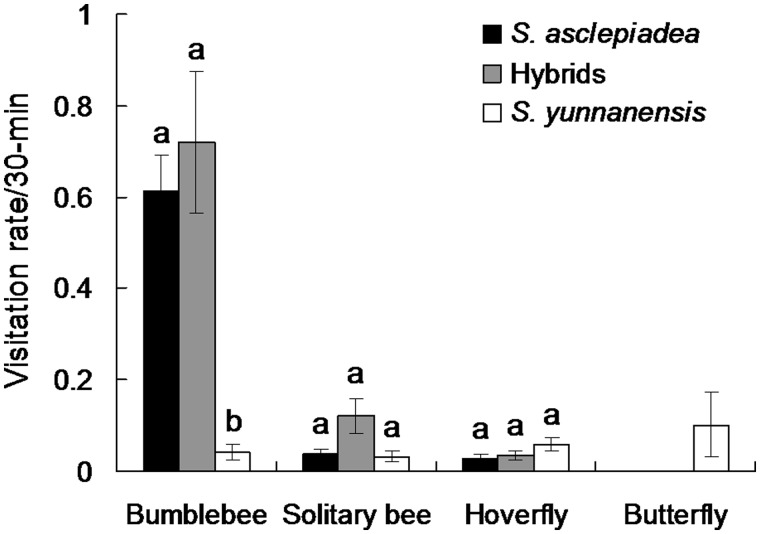
Mean number of visits per flower per 30-min observation period by the four main insect visitors to *S. asclepiadea*, hybrids and *S. yunnanensis*. Letters above bars represent significant differences among species.

We observed totals of 1500, 1129 and 965 flowers for *S. asclepiadea*, *S. yunnanensis* and hybrids, respectively during the 10 h of observations of each group. Bumblebee (Fig. 1) visitation rates were significantly higher to *S. asclepiadea* and hybrids than to *S. yunnanensis* (*F*_2, 57 _=_ _13.196, *P* 0.05) (Fig. 6). Visit rates by solitary bees were significantly higher to hybrids than either parental species (*F*_2, 57 _=_ _4.711, *P* = 0.013). Hoverfly visitation rates to *S. yunnanensis* tended to be higher than to *S. asclepiadea* and hybrids, but rates were not significantly different (*F*_2, 57 _=_ _2.350, *P* = 0.105). Butterflies made mean 0.101 (± 0.072) visits to *S. yunnanensis* but did not visit *S. asclepiadea* or hybrids (Fig. 6). *S.* *asclepiadea* and hybrids were visited primarily by bumblebees, accounting for 77.34 and 63.15 % of the floral visits, respectively, while for *S. yunnanensis*, hoverflies and butterflies were responsible for most visits (36.92 and 30.77 % of visits, respectively).

### Pollen, abortive pollen, ovule and P/O ratio

**Figure 7.**
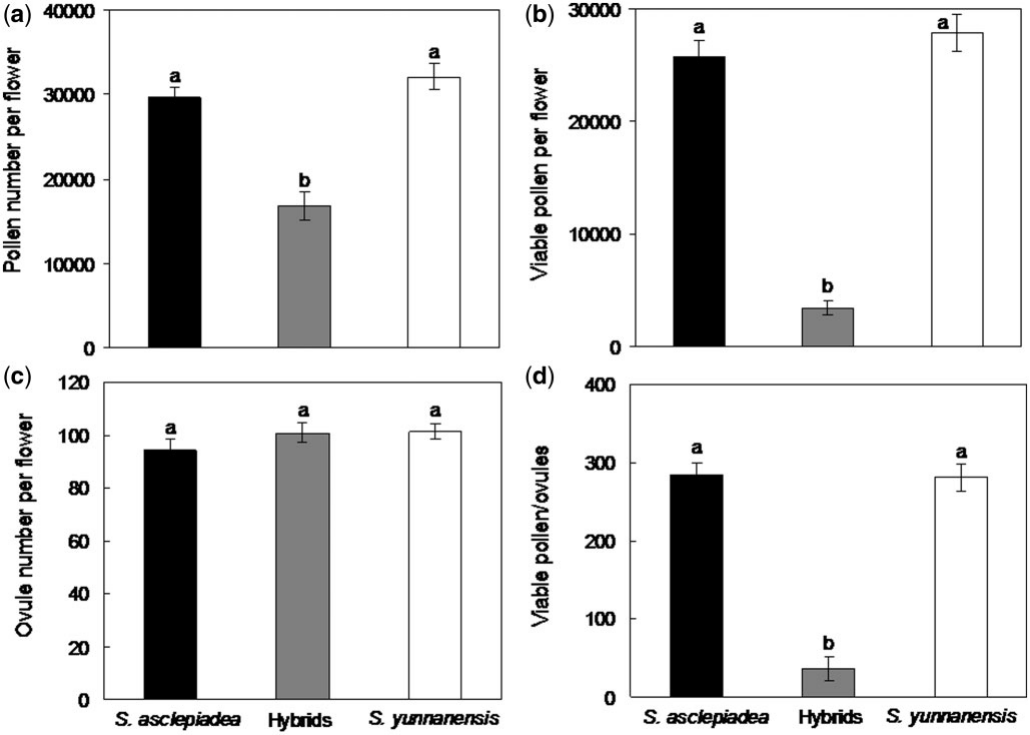
Reproductive parameters (means ± SE) for *S. asclepiadea*, hybrid and *S. yunnanensis*, including **(a)** pollen numbers, **(b)** viable pollen numbers, **(c)** ovule numbers and **(d)** viable P/O. Bars with different letters differ significantly (*P* < 0.05).

Pollen counts between *S. asclepiadae* and *S. yunnanensis* were not significantly different but were significantly higher than those of hybrids (*F*_2, 87 _=_ _30.14, *P* < 0.01); Fig. 7a. Similarly, the number of viable pollen grains per flower was not significantly different between parental species but hybrids produced significantly fewer, <20 % as many viable pollen grains (*F*_2, 87 _=_ _114.41, *P* < 0.01) (Fig. 7b). The number of ovules per bud was not significantly different among the three flower types (*F*_2, 87 _=_ _1.30, *P* = 0.278) (Fig. 7c). Because pollen production but not ovule production was reduced in hybrids, both pollen:ovule ratios (*F*_2, 87 _=_ _271.67, *P* < 0.01) and viable pollen:ovule ratios in hybrids (*F*_2, 87 _=_ _107.60, *P* < 0.01) were significantly lower compared with the parental species (Fig. 7d).

### Fruit/seed set, herbivore ratio and seed mass under natural pollination

**Figure 8.**
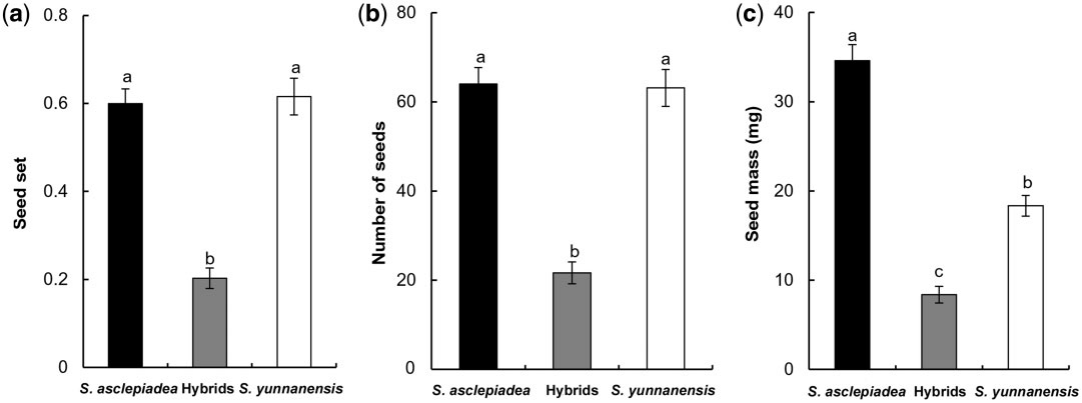
Fecundity measures for *S. asclepiadea*, putative hybrids and *S. yunnanensis* in **(a)** seed set, **(b)** number of seeds and **(c)** seed mass under open pollination. Values indicate means ± SE. Bars with different letters differ significantly (*P* < 0.05).

Fruit set of open-pollinated flowers averaged 68.56 % (± 2.35 %) in *S. asclepiadea* and 67.74 % (± 2.97 %) in *S. yunnanensis*, and did not differ statistically between them (*F*_1, 58 _=_ _0.047, *P* = 0.829). When compared with *S. asclepiadea* (19.95 ± 3.69 %), predation of developing fruits was significantly higher (*F*_1, 58 _=_ _49.561, *P* < 0.01) in *S. yunnanensis* (63.96 ± 5.04 %). For putative hybrids, the fruit set and percent of fruits exhibiting herbivory were 52.21 % (± 6.58 %) and 17.35 % (± 8.93 %), respectively. We did not compare the differences between putative hybrids and parental species for fruit set or percent of fruit damaged by herbivores because small sample sizes for the hybrids limited statistical power. Seed set between *S. asclepiadea* and *S. yunnanensis* was not significantly different, but was significantly higher than in the putative hybrids (*F*_2, 87 _=_ _47.63, *P* < 0.01) (Fig. 8a). Similarly, the putative hybrids produced significantly fewer seeds per fruit than did *S. asclepiadea* and *S. yunnanensis* (*F*_2, 87 _=_ _48.31, *P* < 0.01) (Fig. 8b). Average seed mass significantly differed (*F*_2, 87 _=_ _95.92, *P* <  0.01), with the mass highest in *S. asclepiadea*, intermediate in *S. yunnanensis* and lowest in the putative hybrids (Fig. 8c).

## Discussion

### Interspecific hybridization evidence

Previous studies suggest that interspecific hybridization is of relatively frequent occurrence among species of *Silene* (Karrenberg and Favre 2008; Kruckeberg 1955; Mitchell and Uttal 1969; Minder *et al.* 2007) and historic hybridization events between moderately distant species were significant in generating several *Silene* species (Petri *et al.* 2013). Although several *Silene* species are sympatric in Asia, particularly in mountain areas in southwest China, this is the first report of natural hybridization. We used eight microsatellites markers and two chloroplast sequences to test the hypothesis of potential hybridization between *S. asclepiadea* and *S. yunnanensis*. We found both morphological and molecular evidence that hybridization has occurred between the two species in an area of sympatry. Based on morphological characters, putative hybrids were intermediate to the parental species for four of five morphological quantitative characteristics but similar to *S. asclepiadea* species for one of the quantitative characteristics. In addition, individuals with intermediate flower and fruit traits were identified by PCA analysis (Fig. 2), suggesting hybridization has occurred. Moreover, all microsatellite alleles at the surveyed loci in the putative hybrids occur in the parental taxa, suggesting that the hybrids are not a well differentiated separate lineage. In addition, three species-diagnostic microsatellites markers for *S. asclepiadea* and *S. yunnanensis* (sl-eSSR08, sl-eSSR12 and sl-eSSR17) were detected in all the putative hybrids (Table 1). Moreover, all putative hybrids had chloroplast *psbA*/*trnH* and *atpB*/*rbcL* sequences identical with *S. asclepiadea*, suggesting that *S. asclepiadae* has been the ovule parent when hybridization has occurred*.* Subsequent work should investigate other contact zones to determine whether the pattern of asymmetric hybridization is consistent.

Multiple prezygotic and post-zygotic reproductive barriers contribute to isolate potentially interfertile species (Grant 1949; Rieseberg and Willis 2007). Flowering asynchrony or pollinator isolation may act as pre-mating isolating mechanisms. *S.* *asclepiadea* and *S. yunnanensis* have partially overlapping flowering periods and share some pollinators, suggesting incomplete prezygotic isolation and a possibility of interspecific hybridization. Moreover, our artificial reciprocal crossings between *S. asclepiadea* and *S. yunnanensis* confirmed that the two species were cross-compatible and pollen and ovule counts from putative hybrids indicated that hybrids were fertile, suggesting post-zygotic isolation is also incomplete. The present results indicate that the natural hybridization has occurred between *S. asclepiadea* and *S. yunnanensis.*

### Asymmetric hybridization between *S. asclepiadea* and *S.* *Yunnanensis*

The cpDNA type of 11 putative hybrids matched that of *S. asclepiadea* (H1 and H3) and the PCA analyses placed the hybrids in an intermediate position between *S. asclepiadea* and *S. yunnanensis*, suggesting that *S. asclepiadea* was the female parent and the paternal parent was *S. yunnanensis*. If the trend continues, the direction of hybridization should be unidirectional. Asymmetric hybridization is relatively common in plants (Minder *et al.* 2007; Wu and Campbell 2005; Zha *et al.* 2010; Muranishi *et al.* 2013), which can be explained by a variety of causes, including unilateral incompatibility, as well as differences in phenology, pollinator preferences and local abundance of parental species in contact zones (Carney *et al.* 2000; Muranishi *et al.* 2013; Zha *et al.* 2010).

Asymmetric hybridization can be influenced by the local abundance of parental taxa (Burgess *et al.* 2005; Carney *et al.* 2000; Lepais *et al.* 2009), i.e. there is a tendency for locally rare species to provide the female parent in interspecific hybridization. However, the evidence for such an effect is often incomplete. Our field survey found both parental species had similar local abundances in the studied natural community. Thus, the uneven abundance of parental taxa can be ruled out as a cause for asymmetry in this case.

Temporal asynchronism of the flowering contributes to asymmetric hybridization. Both study species are protandrous and peak flowering of *S. asclepiadae* precedes that of *S. yunnanensis* by about ten days. Consequently, late in the flowering of *S. asclepiadae*, there are likely flowers lacking a conspecific pollen source but with male-phase *S. yunnanensis* in the vicinity. The flowering phenologies of putative hybrids and *S. yunnanensis* are strikingly similar. This similarity should allow for more opportunities for hybrids to backcross with *S. yunnanensis*, perhaps contributing to the greater genetic contributions of *S. yunnanensis* to the hybrids according to the microsatellite data; conversely, more backcrossing with *S. yunnanensis* may explain why the flower phenology of hybrids and *S. yunnanensis* are closely matched. Thus, this unilateral pre-zygotic barrier may be attributable to asymmetric hybridization.

Pollinator preference has been implicated as promoting asymmetrical hybridization between species. In animal-pollinated plant species, pollen movement depends on both pollinator behaviour and effectiveness. Thus, selection on floral traits by pollinators is expected to play a key role in the occurrence of asymmetric hybridization (Zha *et al.* 2010). Our results indicated butterflies were less common pollinators and only visited *S. yunnanensis*. Moreover, although bumblebees, solitary bees and hoverflies visit both parental species, the hoverflies and solitary bees are likely less effective pollinators. Based on our field investigation, we found that most visits to *S. asclepiadea* were bumblebees, which showed a stronger preference for *S. asclepiadea* (Fig. 4), meaning that the visitation pattern would result in conspecific pollen receipt as bumblebees had higher pollinator efficiency than that of hoverflies and solitary bees for most species. Furthermore, pollen grains of *S. yunnanensis* were observed on the stigmas of *S. asclepiadea*, but no pollen of *S. asclepiadea* was on the stigmas of *S. yunnanensis* for three consecutive years (Gao 2015). Pollinator preference or behaviour resulting in asymmetrical pollen flow has been also observed within the genera *Mimulus* (Schemske and Bradshaw 1999), *Nicotinana* (Ippolito *et al.* 2004), *Iris* (Matin *et al.* 2008) and *Rhinanthus* (Natalis and Wesselingh 2012). These results suggest that pollinator preference may partly explain the asymmetric hybridization between *S. asclepiadea* and *S. yunnanensis*.

### The maintenance of species boundaries

*Silene* *asclepiadea* and *S. yunnanensis*, two closely related species that coexist in southwest China, provide insight into the mechanisms underlying the maintenance of species boundaries in hybrid zone. Despite the potential for interspecific hybridization between *S. asclepiadea* and *S. yunnanensis*, the present study shows that individuals with intermediate morphological phenotypes are rare and many microsatellite alleles were not shared between the two species. Thus, *S. asclepiadea* and *S. yunnanensis* have maintained their species boundaries in sympatry despite the potential for gene exchange via natural hybridization.

Ecological and floral isolation are two important factors in preventing hybridization in plant species. Goulson (2009) proposed the species boundary between *Silene dioica* and *S. latifolia* was probably maintained primarily by strong selective forces associated with habitat. There were not apparent differences in either light intensity or relative edaphic conditions between the microhabitats of parental species at the study site (Zhang *et al.* pers. obs.), but broader survey of the habitats occupied by the parental species would help to determine more definitively whether ecological differentiation contributes to the species boundary. Considering the significant differences in floral morphologies between *S. asclepiadea* and *S. yunnanensis*, floral isolation may account for maintenance of their species boundary despite some hybridization occurring. Although some pollinator groups visited putative hybrids and both parental species, bumblebees, the highest visit rates of all pollinators in this study, showed clear preferences for *S. asclepiadea* and hybrid plants compared with *S. yunnanensis*, whereas the butterflies visited only *S. yunnanensis*. Pollinator preference resulting from selection on floral traits by pollinators may contribute to the maintenance of species boundaries in sympatry (Brothers and Atwell 2014). Our findings provide further empirical evidence that floral traits may contribute to maintenance of species boundaries.

Low fitness often occurs in hybrids as a consequence of genomic incompatibility between the parents or disruption of co-adapted complexes (Rieseberg *et al.* 2003). Relatively low fitness of hybrid progeny, suggesting a reduced competitive ability, may play a significant role in preventing introgression. In the present study, the quantity of viable pollen grains and the viable pollen ratio were significantly lower in putative hybrids than in parents. In addition, we found the seed set of putative hybrids was approximately one-third that of parental species, and the seed mass of putative hybrids was considerably less than that of parent species. Thus, putative hybrids had a lower average relative reproductive performance at early life stages than their parents. Reduced seed production could be due to low pollination success or reduced success in producing seeds. Given that the putative hybrids received high visit rates and that they produced less pollen, it seems more likely that reduced seed production relates to an inability to mature seeds rather than to inadequate pollen receipt. Our analyses do not allow determination of the ancestry of the putative hybrids (e.g. F1, F2, backcross etc.), and it is possible that reproductive output differs among these different generations. Other studies also indicated that hybrids had intermediate or inferior performance compared with parent species (Fritz *et al.* 2006; Ross *et al.* 2012). Reduced pollen and seed production observed in putative hybrids may contribute to maintenance of the species boundary of *S. asclepiadea* and *S. yunnanensis.*

## Conclusions

Based on the molecular and morphological data, we confirmed that interspecific hybridization occurred between *S. asclepiadea* and *S. yunnanensis*. The direction of hybridization is asymmetric from *S. yunnanensis* to *S. asclepiadea*. Phenology, a unilateral pre-zygotic barrier, and pollinator preference may have been responsible for the asymmetric hybridization observed. However, our field investigation indicated *S. asclepiadea* and *S. yunnanensis* were common and the hybrids were rare in the community. Thus, *S. asclepiadea* and *S. yunnanensis* can maintain species boundaries despite ongoing hybridization. Floral isolation and low viable pollen and seed production of the putative hybrids contribute to maintenance of species integrity in sympatry. In the present study, our results only focused on early life stages. Future studies including collecting the data about the fitness at later life stages and enlarging sample sizes and studied sites are necessary to further evaluate the mechanisms of species boundaries in spite of inter-hybridization in sympatry. 

## Sources of Funding

This research was supported by a postdoctoral research Foundation grant 2013M540600 to J.-J.Z., a Research Fellowship for International Young Scientists by NSF of China (grant no. 30910362) to B.M. and NSFC (grants no. 31030016, U1402267) to S.-Q.H. 

## Contributions by the Authors

J.-J.Z. and B.R.M. collected data from the field populations. J.-J.Z. and S.-Q.H. wrote the manuscript. All authors contributed in experimental design, data analysis and commented the manuscript.

## Conflict of Interest Statement

None declared.
